# Supplementary material for: Development and Validation of a Food Frequency Questionnaire for Evaluating the Nutritional Status of Patients with Serious Mental Illnesses (DIETQ-SMI) in Bahrain
Source: Brain Sci. 2024 Mar 26;14(4):312. doi: 10.3390/brainsci14040312 (PMC11047868; doi:10.3390/brainsci14040312)
Supplement: Supplementary file 1 [file brainsci-14-00312-s001.zip › Supplemental Material S1.pdf]

Supplement Material S1

GR: 1) Breads - white, wheat, sourdough, rye, multigrain; 2) Bagels - plain, sesame, poppyseed, cinnamon raisin, blueberry; 3) Rolls - dinner rolls, hamburger buns, hot dog buns; 4) Tortillas - flour, corn, whole wheat; 5) Cereals - bran flakes, corn flakes, crispy rice, granola; 6) Oatmeal - regular, steel cut, instant; 7) Grits - plain, cheese, bacon; 8) Porridge - regular, steel cut, creamy; 9) Rice - white, brown, jasmine, basmati, wild; 10) Pasta - spaghetti, angel hair, penne, rotini, spirals; 11) Noodles - egg noodles, soba, udon, ramen; 12) Couscous or Hareesa - plain, flavored; 13) Crackers - saltines, Ritz, Triscuits, Wheat Thins, graham; 14) Pretzels - plain, salted, sticks, flavored twists; 15) Popcorn - plain, buttered, flavored; 16) Pancakes - buttermilk, blueberry, chocolate chip, banana; 17) Waffles - plain, blueberry, chocolate chip, pecan; 18) French toast - classic, cinnamon, strawberry, whole wheat; 19) Muffins - blueberry, bran, banana nut, corn; 20) Donuts - glazed, chocolate, jelly filled, powdered; 21) Granola bars - chewy, crunchy, nut, seed, chocolate.

FR: 22) Apples - Red Delicious, Gala, Granny Smith, Fuji, Honeycrisp; 23) Bananas - regular, plantain; 24) Oranges - navel, Valencia, blood orange, mandarin; 25) Grapes - green, red, purple, seedless, cotton candy; 26) Strawberries - fresh, frozen; 27) Blueberries - fresh, frozen, dried; 28) Raspberries - fresh, frozen; 29) Blackberries; 30) Cherries - sweet, sour; 31) Cantaloupe; 32) Honeydew; 33) Watermelon; 34) Peaches - yellow, white; 35) Plums - red, black, green; 36) Apricots - fresh, dried; 37) Pineapple - fresh, canned, juice; 38) Mango - fresh, dried; 39) Kiwifruit - green, golden; 40) Pears - green, red, Asian; 41) Fruit juices - orange, apple, grape, cranberry, pineapple, prune.

VEG: 42) Lettuce - romaine, iceberg, green leaf, red leaf, spring mix; 43) Spinach - fresh, frozen, canned; 44) Kale - curly, Tuscan; 45) Arugula; 46) Tomatoes - cherry, grape, heirloom, beefsteak; 47) Carrots - regular orange, rainbow baby; 48) Broccoli; 49) Cauliflower; 50) Potatoes - Russet, red, yellow, fingerling; 51) Sweet potatoes - orange, purple; 52) Corn - on the cob, kernels, creamed; 53) Peas - fresh, frozen; 54) Green beans; 55) Lima beans; 56) Onions - yellow, white, red, green; 57) Garlic - minced, roasted; 58) Mushrooms - white, portobello, shiitake; 59) Celery; 60) Cucumber; 61) Zucchini; 62) Eggplant; 63) Avocado; 64) Peppers - green, red, yellow, orange; 65) Cabbage - green, purple, savoy; 66) Brussels sprouts; 67) Asparagus; 68) Beets; 69) Okra; 70) Vegetable juices - tomato, carrot, green.

DRY: 71) Milk - whole, 2%, 1%, skim, lactose-free; 72) Soy milk; 73) Almond milk; 74) Yogurt - Greek, regular, low-fat, nonfat, fruit flavored; 75) Cottage cheese - small curd, large curd, low-fat, nonfat; 76) Cheese - cheddar, mozzarella, swiss, provolone, feta; 77) Cream cheese - regular, light, whipped; 78) Ice cream - vanilla, chocolate, strawberry, coffee; 79) Frozen yogurt - vanilla, chocolate, strawberry.

MP: 80) Chicken breast, thighs, drumsticks, wings, ground; 81) Turkey - breast, ground; 82) Beef - ground, steak - sirloin, ribeye; 83) Pork - chops, roast; 84) Lamb - chops, leg; 85) Fish - seabass, sea bream, safi, salmon, tuna, cod, tilapia, shrimp; 86) Eggs - scrambled, fried, boiled, poached; 87) Tofu - soft, firm, baked, fried; 88) Tempeh - plain, flavored; 89) Almonds, cashews, peanuts, walnuts, pecans; 90) Sunflower seeds, pumpkin seeds; 91) Nut butters - peanut, almond, sunflower seed; 92) Beans - black, pinto, kidney, cannellini, chickpeas; 93) Lentils - red, brown, green; 94) Seitan.

OFI: 95) Olive oil - extra virgin, regular; 96) Canola oil; 97) Vegetable oil - soybean, corn; 98) Coconut oil; 99) Butter - salted, unsalted; 100) Margarine - stick, tub, liquid squeeze; 101) Mayonnaise; 102) Salad dressing - ranch, Italian, balsamic vinaigrette; 103) Avocado; 104) Olives - green, black, Kalamata; 105) Bacon; 106) Sausage - pork, chicken, turkey.

BEV: 107) Water - tap, bottled, sparkling, flavored; 108) Coffee - black, with cream and sugar; 109) Iced coffee; 110) Tea - black, green, herbal (peppermint, chamomile); 111) Iced tea - sweet, unsweetened; 112) Soda - regular, diet; 113) Energy drinks; 114) Fruit juice - orange, apple, grape; 115) Vegetable juice - tomato, carrot, green; 116) Milk - whole, 2%, skim; 117) Chocolate milk; 118) Milk alternatives - almond, soy, oat, rice; 119) Smoothies - fruit, green, protein; 120) Lemonade, fruit punch; 121) Beer - lager, ale, stout, wheat; 122) Wine - red, white, rose, sparkling; 123) Cocktails - margarita, martini, vodka tonic, whiskey sour; 124) Hot chocolate; 125) Lattes - hot, iced; 126) Frozen blended coffee drinks.

SSS: 127) Potato chips - regular, flavored; 128) Pretzels; 129) Popcorn; 130) Crackers - saltines, graham, Wheat Thins; 131) Cookies - chocolate chip, oatmeal raisin, sugar; 132) Cake - chocolate, vanilla, carrot, pound cake; 133) Pie - apple, cherry, pumpkin, lemon meringue; 134) Cupcakes; 135) Pastries - croissants, danishes, strudels; 136) Donuts; 137) Candy - chocolate, lollipops, gummy bears; 138) Chocolate - bars, chips, truffles; 139) Ice cream - vanilla, chocolate, rocky road, cookies and cream; 140) Frozen yogurt; 141) Sugary cereal - Froot Loops, Cocoa Puffs, Honey Smacks; 142) Granola bars - chewy, crunchy; 143) Pizza; 144) French fries; 145) Hamburger; 146) Hot dog; 147) Deli sandwich - ham, turkey, roast beef; 148) Wraps - chicken, veggie, steak; 149) Sugary drinks - soda, sweet tea, energy drinks.

COND: 150) Ketchup; 151) Mustard - yellow, Dijon, honey; 152) Mayonnaise; 153) Salad dressing - ranch, Italian, balsamic, Caesar; 154) Soy sauce; 155) Hot sauce - Tabasco, sriracha; 156) Barbecue sauce - sweet, smoky, spicy; 157) Salt - table salt, sea salt, kosher salt; 158) Pepper - black pepper, cayenne; 159) Herbs - basil, oregano, cilantro, parsley, rosemary; 160) Spices - cinnamon, nutmeg, ginger; 161) Syrup - maple, corn, chocolate; 162) Honey; 163) Sugar - white, brown, powdered, raw; 164) Sweeteners - stevia, Splenda, Equal, Sweet n Low; 165) Salsa - mild, medium, hot; 166) Guacamole; 167) Hummus - classic, roasted red pepper; 168) Cream cheese - plain, chive, jalapeño; 169) Jam, jelly - grape, strawberry, peach, apricot; 170) Peanut butter - creamy, crunchy; 171) Almond butter; 172) Sunflower seed butter.
