# Supplementary material for: Development and Validation of a Food Frequency Questionnaire for Evaluating the Nutritional Status of Patients with Serious Mental Illnesses (DIETQ-SMI) in Bahrain
Source: Brain Sci. 2024 Mar 26;14(4):312. doi: 10.3390/brainsci14040312 (PMC11047868; doi:10.3390/brainsci14040312)
Supplement: Supplementary file 1 [file brainsci-14-00312-s001.zip › Supplemental Material S2.pdf]

Supplemental Material S2

Mark HOW OFTEN you consumed the following foods during the <u>two weeks</u><br>Caution, answer considering as portion the quantity that is entered in the parenthesis<br>(*t* =times, *g*= gram, pcs =pieces, *c* = cup =240 ml)							
**SN**	**Item**	**Never/ Rarely**	**1-3 t/ month**	**1-2t/ week**	**3-6t/ week**	**1t/ day**	**≥2t/day**
1	White “Lebanese/Arabic” bread (1 pc, 30gr)						
2	White toasted bread (2 pcs)						
3	Brown “Lebanese/Arabic” bread (1 pc, 30gr)						
4	Brown toasted grain bread (2 pcs)						
5	Biscuits (2 pcs), rusks (1 pcs), cookies (2 pcs)						
6	Cereals (1 cup), cereals bar (1 pcs)						
7	Beef (steak) (1p~150 grams)						
8	Burgers (1 pc)						
9	meatballs (4 pcs)						
10	minced meat (1c)						
11	Chicken (all types) (150 gr)						
12	Lamb, goat, deer, rabbit, lamb chops (150 grams)						
13	Fish (approx. 150 g, specify)						
14	Seafood (octopus, squid, shrimp) (150 grams)						
15	Lentils, beans, chickpeas (1 c) (1 dish = 2 cups)						
16	Fresh soup, (1 portion=250ml)						
17	Soup with pasta (e.g. noodles) (1 portion=250ml)						
18	Rice, (1 cup, 1 medium plate)						
19	Boiled potatoes, mashed potatoes (1 medium)						
20	French fries (1 portion)						
21	Pizza (1 slice)						
22	Fresh Fruits (specify) Apples, bananas, oranges						
23	Fresh Fruits (specify) Strawberries, berries, pineapples						
24	Fresh Fruits (specify) Watermelons, melons						
25	Fresh Vegetables (specify) Potatoes, tomatoes, onions						
26	Fresh Vegetables (specify) Carrots, cucumbers						
27	Fresh Vegetables (specify) Sweet Chili, Salads						
28	Dried fruits (¼ cup)						
29	Dried nuts, nuts (¼ cup)						
30	Yoghurt complete or light (1 tub)						
31	Cream cheese “Glasses” (25 gr)						
32	Feta, white cheese, hard cheese (25 gr)						
33	Egg (boiled, fried, omelet) (1 pcs)						
34	Pies (ex. Cheese pie, spinach pie) (1 portion)						
35	Ice cream, milk shake, pudding, rice pudding (1 pcs)						
36	Honey, jam (1 teaspoon)						

37	Olives (10 small /5 large)						
38	Chocolate (all types) (1 medium = 60 gr)						
39	Chips packs, popcorn (1 bag =70 gr)						
40	Fruit juice (1 glass or 1 small juice pack)						
41	Soft drinks, (1 can)						
42	Milk, milk shake (1 glass)						
43	Coffee in a cup (e.g. Americano, Espresso)						
44	Arabic Coffee						
45	Tea, other herbal teas (e.g. chamomile, peppermint) (1 cup)						
46	Isotonic/energy drinks (1 glass)						
47	Alcoholic drinks (wine, beer, whisky, vodka, (1 unit)						
48	Honey						
49	Herbs and Spices (basil, oregano, cinnamon, cumin, and paprika) (1 tea spoon)						
50	Oils and Fats: Oils such as olive oil, vegetable oil, and coconut oil, as well as fats like butter, are used for cooking, frying, and baking (1 spoon).						
